# The structural and the photoelectrochemical properties of ZnO–ZnS/ITO 1D hetero-junctions prepared by tandem electrodeposition and surface sulfidation: on the material processing limits†

**DOI:** 10.1039/c8ra00176f

**Published:** 2018-03-27

**Authors:** A. Brayek, S. Chaguetmi, M. Ghoul, I. Ben Assaker, R. Chtourou, P. Decorse, P. Beaunier, S. Nowak, F. Mammeri, S. Ammar

**Affiliations:** ITODYS, Université Paris Diderot, USPC, CNRS UMR-7086 Paris France ammarmer@univ-paris-diderot.fr; Lab. Photovoltaïque, Centre de Recherches et des Technologies de l'Energie Technopole Borj Cedria, Faculté des Sciences Hammamm lif Tunisia; Université 20 Août 1955 de Skikda Skikda Algeria; Sorbonne Université, CNRS, Lab. de Réactivité de Surface, LRS Paris France

## Abstract

ZnO–ZnS 1D hetero-nanostructures were prepared by an easy and scalable processing route. It consists of ZnO nanorod electrodeposition on ITO substrate and surface sulfidation by ion exchange in an aqueous Na_2_S solution. Increasing the treatment contact time (*t*_c_) from 8 to 48 h involves different ZnS growth mechanisms leading to different structural and microstructural rod characteristics, even if the overall size does not change significantly. Grazing X-ray diffraction, high-resolution microscopy, energy-dispersive spectrometry and X-ray photoelectron spectroscopy describe the outer surface layer as a poly- and nanocrystalline ZnS blende shell whose thickness and roughness increase with *t*_c_. The ZnO wurtzite–ZnS blende interface goes from continuous and dense, at short *t*_c_, to discontinuous and porous at long *t*_c_, indicating that ZnS formation proceeds in a more complex way than a simple S^2−^/O^2−^ ion exchange over the treatment time. This feature has significant consequences for the photoelectrochemical performance of these materials when they are used as photoanodes in a typical light-assisted water splitting experiment. A photocurrent (*J*_p_) fluctuation of 45% for less than 5 min of operation is observed for the sample prepared with a long sulfidation time while it does not exceed 15% for that obtained with a short one, underlining the importance of the material processing conditions on the preparation of valuable photoanodes.

## Abstract 2

ZnO nanorods were electrodeposited on ITO and immersed in a Na_2_S solution for a variable time. As a function of this experimental parameter, different ZnS surface growth mechanisms take place, leading to specific microstructures.

## Graphical abstract

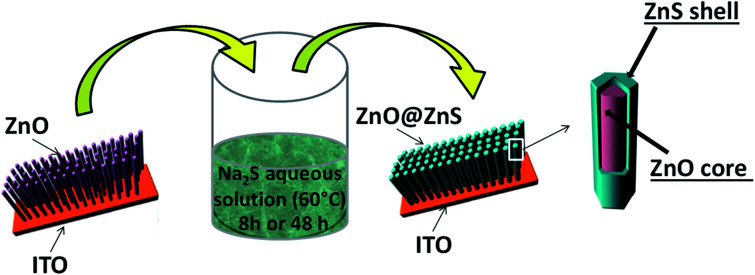

## Introduction

Semiconducting core–shell hetero-nanostructures have been the focus of functional materials science and engineering. Their synergetic optoelectronic properties make them particularly suitable for light-sensitive devices. Nowadays, with optimized architectures, they are seriously considered for photo-electrochemical (PEC) water splitting. Indeed, when the relative positions of the conduction and valence bands of the core and shell semiconductors are adjusted to straddle the chemical potentials of the H_2_/H^+^ and OH^−^/O_2_ water redox reactions, the resulting heterostructures can be employed as anodes for water oxidation, under sunlight illumination.

In a typical PEC cell, this reaction is accompanied by water proton reduction at the counter-electrode, a Pt wire or sheet, leading to molecular hydrogen generation. These hybrids, when they are well designed and produced with high crystalline quality, harvest sunlight efficiently, create the desired electron–hole pairs and separate them quickly, avoiding their recombination. Most of the photogenerated holes are transferred to the valence band of the shell semiconductor, and are released to the electrolyte solution to be involved in the water oxidation reaction, while most of the photogenerated electrons are concentrated into the conduction band of the core semiconductor to be injected into the external circuit and then consumed in water reduction at the surface of the counter-electrode.^1^

ZnO (band-gap = 3.39 eV) and ZnS (band-gap = 3.60 eV) are two direct wide-band gap semiconductors. In one hand, their conduction and valence band-edges straddle the water-redox levels, and in the other hand, their relative band-energy positions offer a favorable charge separation. The energy positions of the conduction and valence bands of ZnS are higher than those of ZnO.^2,3^ This means that holes and electrons generated by ZnS and ZnO in a PEC experiment can be collected mainly in the valence band of ZnS and the conduction band of ZnO, respectively.

Already ZnO–ZnS-based core–shell-type nanostructures have been explored by different groups for such a purpose. Several material processing methods have been developed to produce them with a special emphasis on 1D-vertically oriented architectures. The interest to such a morphology is mainly due to is ability to enhance light scattering and promote multiple absorptions compared to other morphologies.^4–7^

It must be noticed that the preparation of ZnO–ZnS nanorods, as individual objects or as dense arrays, is not at all trivial. It is important to control the chemical and physical interfacing of the constituting semiconductors. A weak control means a high interfacial structural defect density and then a high charge trapping probability (the traps act mainly as non-radiative recombination sites), reducing significantly the photoconversion yield.^8–10^

Recently, ion exchange was proposed as a facile and effective method for the preparation of ZnO–ZnS heterostructures, starting from preformed ZnO nanostructures.^10–14^ In practice, ZnO rods are immersed in a concentrated aqueous solution of Na_2_S (typically 0.1–1.0 M) at room or moderate temperature (commonly ≤70 °C) and stirred to allow ZnS shell growth for various contact times (usually 8–12 h).^10–14^ The general ZnO to ZnS transformation scheme is assumed to be a progressive replacement of O^2−^ lattice anions by solvated S^2−^ ones, through a double ion-diffusion pathway: O^2−^ from the solid to the solution and reversely S^2−^ from the solution to the solid, where the general morphology of the starting ZnO particles is maintained. As a consequence, the longer the contact time (*t*_c_) is, the thicker the formed ZnS shell becomes. The thicker the formed ZnS shell is, the better PEC performances are.^14,15,33^

Focusing on this material processing route, we wanted to explore its limits for the preparation of efficient ZnO–ZnS based PEC photoanodes. In practice, we applied it, varying the experimental synthesis conditions, mainly *t*_c_, in order to prepare different ZnO–ZnS 1D-arrays and we evaluated their PEC response in relation to their microstructure. Typically, we immersed ZnO nanorods (NRs), deposited as a dense array on a conductive Indium doped thin oxide film (ITO), in a Na_2_S solution for a short (8 h) and long (48 h) contact times. We denoted the bare ZnO NRs and their related ZnO–ZnS composites as ZO, ZOS8 and ZOS48, respectively. We measured their PEC properties, working within a home-made three-electrode cell and using a passive Na_2_SO_4_ electrolyte solution (0.5 M, pH 7). We discussed then all the obtained results in the framework of our main goal.

## Results and discussion

### A Structural and microstructural properties

**Fig. 1:**
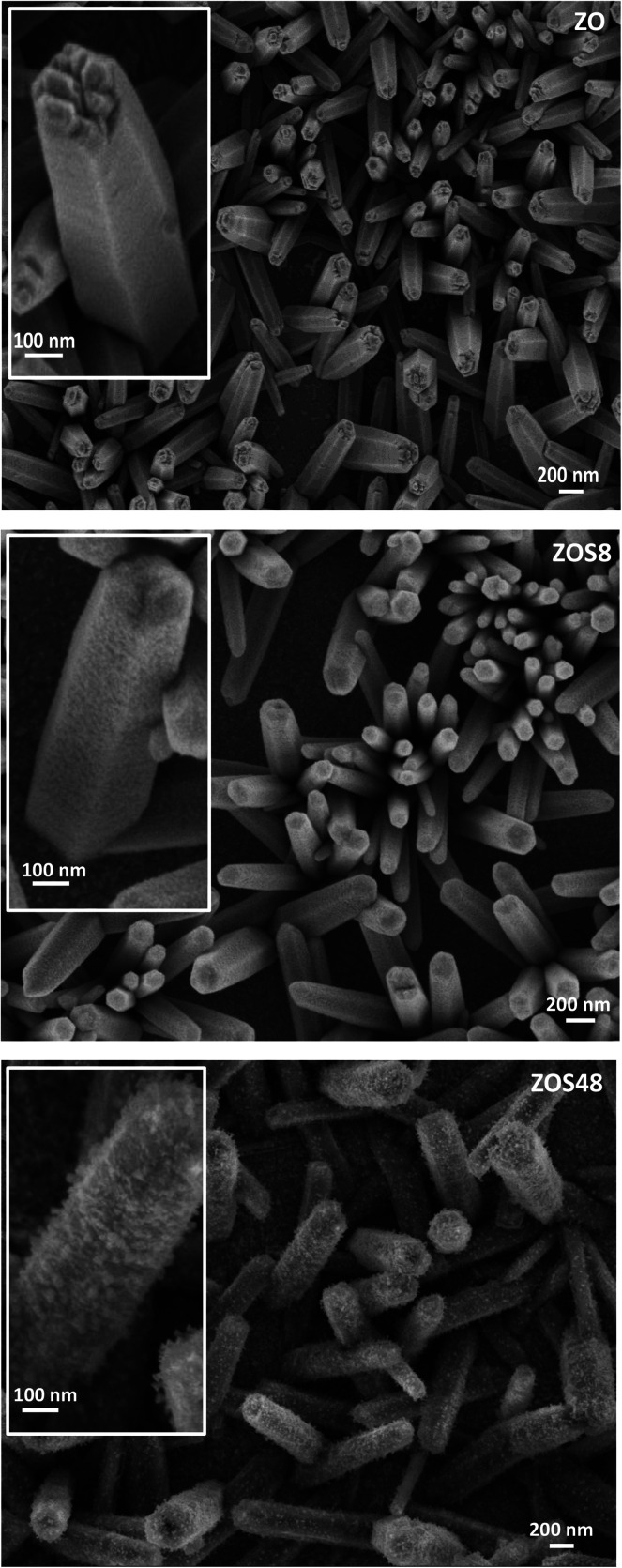
SEM top views of assemblies of ZO, ZOS8 and ZOS48 NRs. A zoom on a representative rod is given in inset for each sample.

**Fig. 2:**
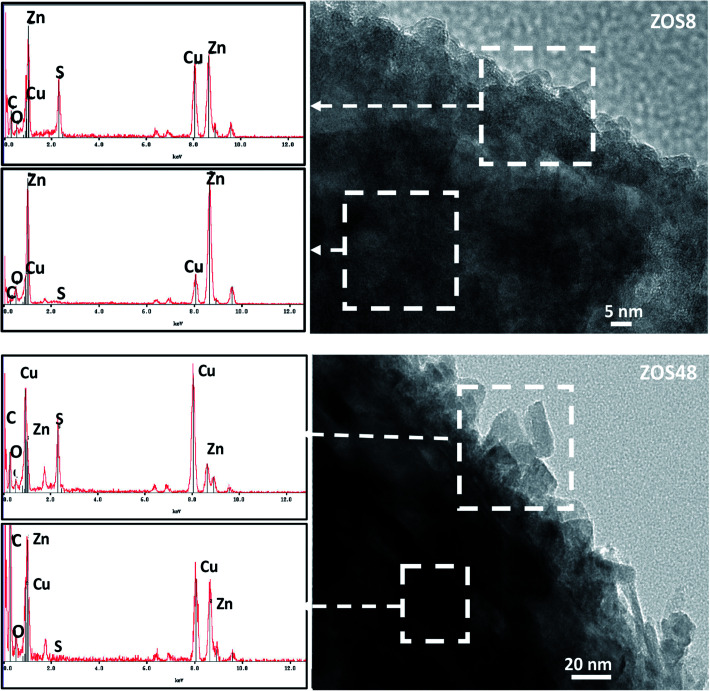
EDS spectra recorded on the outer and inner ZnO–ZnS NR regions for ZOS8 and ZOS48 NRs.

ZnO–ZnS NRs of 10–15 μm long and 100–200 nm in diameter were grown directly on ITO substrates by electrodeposition, followed by sulfidation.^11,15^ The formation of a continuous ZnS shell was confirmed by Transmission and Scanning Electron Microscopies (TEM and SEM) coupled to Energy-Dispersive Spectrometry (EDS). So, the recorded SEM micrographs are found to be typical of fairly dense and well-ordered similarly-sized rods on the ITO surface (Fig. 1). The comparison of the general rod morphology before and after sulfidation reveals changes in the aspect of the external rod surface. The native ZnO NRs have a hexagonal cross-section with a smooth surface whereas the treated ones appear to be more rounded and rough, particularly those corresponding to a treatment time of 48 h. TEM images evidence a contrast difference between the border and the core of some representative composite rods, while they do not for bare ZO ones. Focusing on the chemical composition of the composite samples, EDS-TEM analysis clearly shows that their constituting rods are composed of Zn, O and S atoms, the S signal being mainly confined within the outer region (Fig. 2), in agreement with a core–shell structure.

**Fig. 3:**
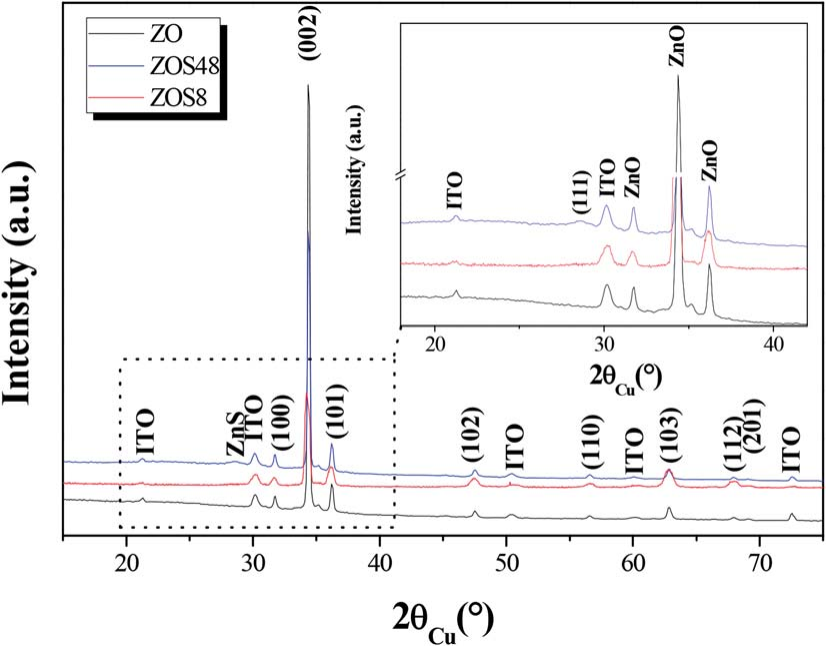
GXRD patterns of ZO, ZOS8 and ZOS48. A zoom on the 18–42° 2*θ* range is given in inset to show the (111) ZnS blende diffraction line.

To complete our investigations, we recorded the X-ray diffraction (XRD) patterns of all samples under grazing conditions (Fig. 3). All appeared to be mainly consistent with the wurtzite ZnO (JCPDS no. 98-005-7478) and the In_1.9_Sn_0.1_O_3_ (JCPDS no. 98-005-0849) phase structures. The measured cell parameters, using Rietveld refinements (Highscore software from PANALYTICAL), were found to be very close to those of bulk ZnO and In_1.9_Sn_0.1_O_3_, respectively (Table 1), suggesting the weakness of the residual strains on the zincite phase due to the ITO substrate and/or ZnS coating on the rods.

**Table 1:** Main structural and microstructural parameters of ZO, ZOS8 and ZOS48 samples as inferred from Rietveld refinement. The fit quality is expressed by the GOF parameter which found to be ranging between 2.2 and 1.8

Phase	Parameters	Samples		
		ZO	ZOS8	ZOS48
ZnO	*a* (Å)	3.24775	3.24721	3.24752
	*c* (Å)	5.20311	5.20373	5.20108
	〈*ε*〉 (%)	0.12	0.22	0.13
	*T*_(002)_	5.37		5.02
	*T*_(100)_	0.10		0.19
ZnS	*a* (Å)	—	a	5.39846
	〈*ε*〉 (%)	—	a	a
ITO	*a* (Å)	10.17818	10.17926	10.17063
	〈*ε*〉 (%)	0.34	0.39	0.33

a Not determined due to lack of reflexions.

The second interesting piece of information inferred from XRD concerns the crystallographic signature of ZnS phase. It is only apparent in the ZOS48 pattern (see the inset in Fig. 3), in agreement with the formation of a thicker ZnS shell. In this pattern a weak and broadened peak appears at 2*θ* = 28.62° corresponding to the ZnS blende (111) diffraction line (JCPDS no. 98-006-0378).

A careful inspection of the data reveals additional information. Microstructural differences between the two composite samples and between them and their ZO parent can be clearly pointed out. Indeed, a net preferential orientation along the wurtzite [002] direction is observed for the ZnO phase but, surprisingly, the intensity enhancement of its related (002) line is less significant in the ZOS8 pattern than in those of ZO and ZOS48. Typically, the calculated texture coefficient T_(002)_/T_(*hkl*)_ of ZOS8 ten time smaller than that of ZO or ZOS48 (Table 1).

**Fig. 4:**
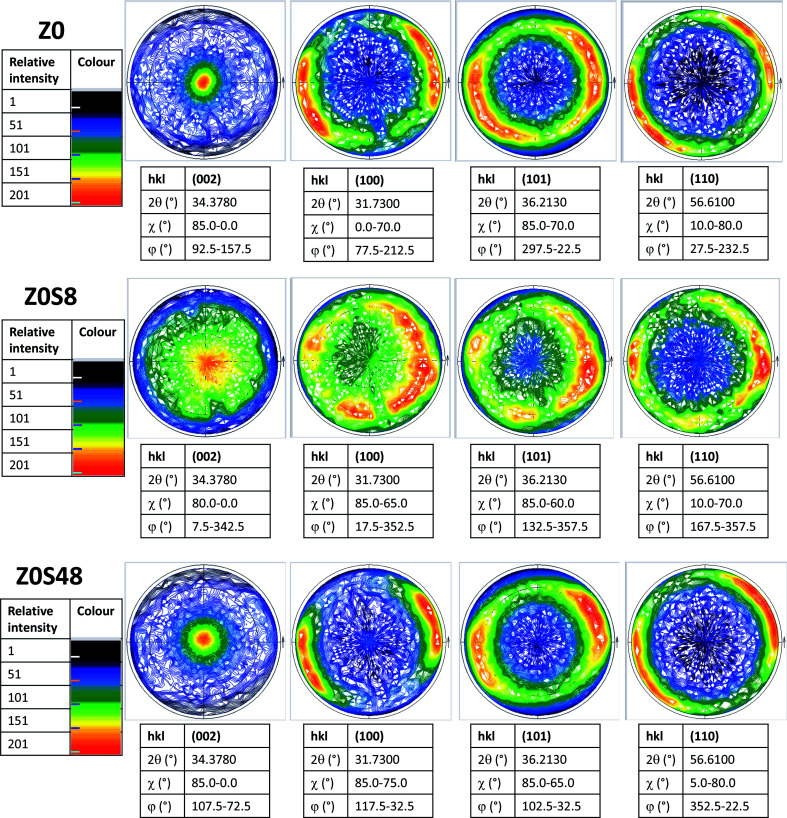
Corrected X-ray pole figures of the ZnO phase recorded on the (002), (100), (101) and (110) crystallographic planes, rotating out- (*φ* rotation) and in- (*χ* tilt) the substrate surface plane, for all samples studied. The figure centre corresponds to the normal direction of the substrate surface.

This feature was also confirmed by recording the X-ray pole figures of the wurtzite phase along different crystallographic directions: perpendicular to the *c* direction (taken parallel to the substrate normal), parallel to it and in between (see Fig. 4 and SI-1 in the ESI†). All the (002) pole figures exhibit a maximum intensity at their centre (*χ* = 0°), meaning that the ZnO crystals are mainly parallel to the substrate surface, but this maximum is less marked for the ZOS8 sample. Moreover, whereas the (101), (102) and (110) pole figures have a net maximum intensity at *χ* ∼ 65, 45 and 85°, for ZO and ZOS48, very close to the tabulated crystallographic angles between the (101), (102) and (110) wurtzite planes and the (002) one, namely 61.6, 43.0 and 90.0°, respectively, these maxima are once again less marked for ZOS8. These results agree with the fact that ZnO grows almost vertically at the surface of ITO, along its *c* lattice axis, and forms crystallites almost all oriented within a solid angle smaller than 90° about the normal of the substrate, but they also indicate the installation of crystallographic disorder on the wurtzite phase during the first hours of sulfidation.

This disorder is also pointed out by a greater ZnO diffraction line broadening associated with a larger average micro-strain-induced deformation 〈*ε*〉 value in the pattern of ZOS8 compared to those of ZO and ZOS48. It is twice as high for ZOS8 as for ZO and ZOS48 (Table 1), suggesting that the formation of the ZnS shell affects the structure of the native ZnO rods mainly at the beginning of the treatment. In other words, the disorder, induced by ZnS sell formation is resorbed when the treatment is prolonged up to 48 h.

**Fig. 5:**
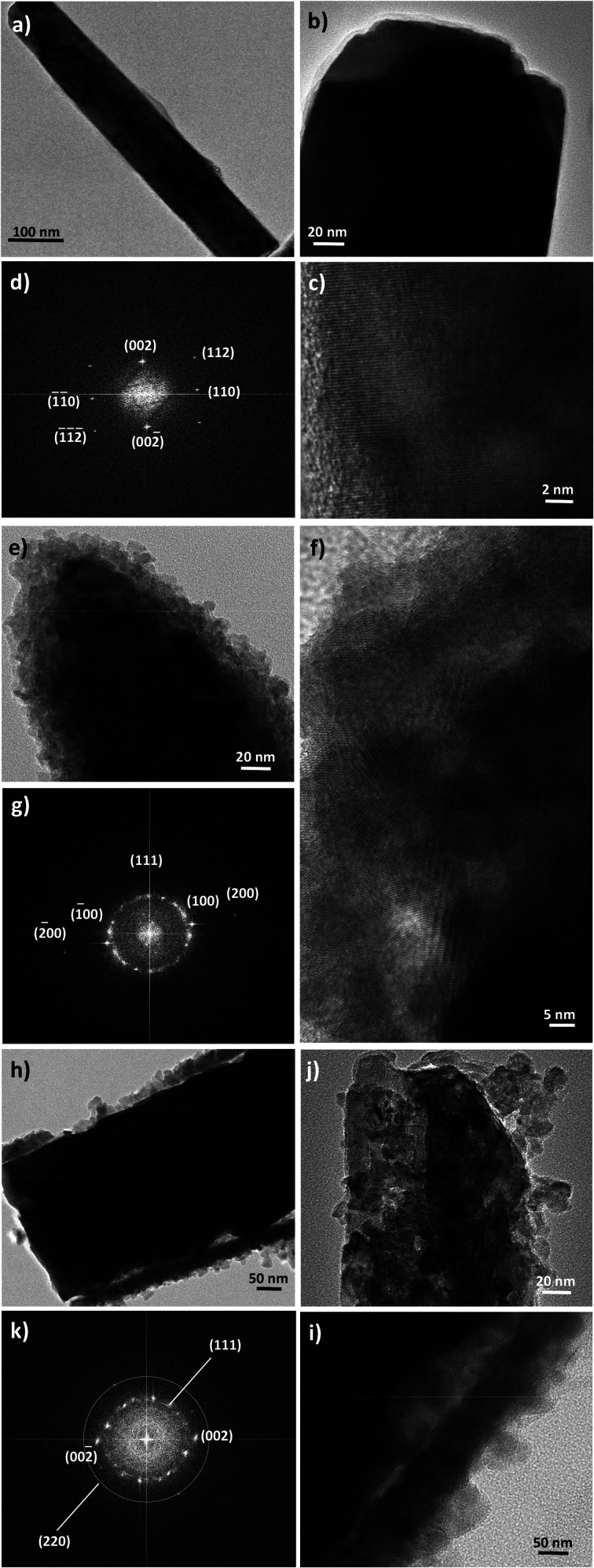
TEM images of representative ZnO rods before (a and b) and after sulfidation for 8 h (e) and 48 h (h and j). Their HRTEM micrographs (c, f and i) and FFTs (d, g and k, respectively).

Finally, high-resolution TEM (HRTEM) observations were performed focusing on the ZnO–ZnS junction (Fig. 5). Interestingly, the rods in ZO are well-defined and free from any coating (Fig. 5a and b). Those in ZOS8 and ZOS48 have a thin coating consisting of a polycrystalline ZnS outer layer, with a roughness which increases with sulfidation time (Fig. 5e and i). The crystals of the shell are 5–8 and 10–13 nm in size in ZOS8 and ZOS48, respectively. These nanocrystals form a more or less continuous layer 15–20 (Fig. 5f) and 40–50 nm (Fig. 5h–j) thick. A dense and continuous layer grows around the ZnO core without any porosity for ZOS8 (Fig. 5e–f), while it has porosities for ZOS48 (Fig. 5h–j), particularly at the ZnO–ZnS interface. The ZnS shell in this sample looks like aggregates of ZnS nanocrystals, rounding the ZnO rods but not always in contact with the ZnO surface. Such a feature suggests the formation of this outer shell through a dissolution–precipitation process.

We suspect that the ZnS shell growth is accompanied by a dissolution of the ZnO core at its interfacial contact region. We believe that the previously identified disoriented ZnO zone is mainly involved in this dissolution process. We believe also that an anarchical precipitation of ZnS crystals occurs from the solution to the surface of preformed ZnS shell, leading to a very porous ZnS outer layer. These hypotheses are supported by other HRTEM observations and analyses. Indeed, the Fast Fourier Transforms (FFT) calculated from the rod surface HRTEM images of the composite samples (Fig. 5g and k) consist of the superposition of a Debye–Scherrer-like pattern, fully indexed in the blende structure, and a Laue-like one, corresponding to the wurtzite structure (Fig. 5g and k).

The distances measured on the main diffraction ring are consistent with the (111) ZnS blende crystallographic plane (3.11 Å). Those measured on the main spots correspond to the (100) ZnO wurtzite crystallographic planes for ZOS8 and to (002) for ZOS48. Once again, a less preferential orientation of the ZnO crystallites along the wurtzite *c* axis in ZOS8 sample is indicated. For this sample, in the rod border zones, the wurtzite (100) planes are much more exposed than the (001) ones. These planes are perpendicular to the ZnO direction of growth, which is assumed to be parallel to the *c* axis, suggesting that ZnS shell formation does not simply consist of anion exchange between O^2−^ and S^2−^ at the rod surface. Anion and cation solid–solid diffusion must proceed in the chemically modified surface zone but also around it, transforming the former into a cubic lattice and maintaining the hexagonal stacking of the latter but reorienting it in favour of a (100) wurtzite and (111) blende crystallographic junction. This reorientation tends to reduce the mismatch between the two lattices. The relative difference between the reticular distance of the blende (111) planes and the wurtzite (100) ones is about 9% while it is higher than 16% between the same blende planes and the wurtzite (002) ones.

In ZOS48 sample, these crystallographically disoriented interfacial wurtzite nanocrystals are absent, and they are replaced by a kind of interfacial porosity, agreeing well with the dissolution of these crystals. The Zn^2+^ cations, lost during this dissolution, are assumed to be consumed by reacting with dissolved S^2−^ anions and contribute to the anarchical precipitation of ZnS crystals on the outer rod's surface. In this scenario, the preferential orientation of the wurtzite phase along the *c* axis is recovered and the polycrystallinity of the blende phase becomes more marked.

**Fig. 6:**
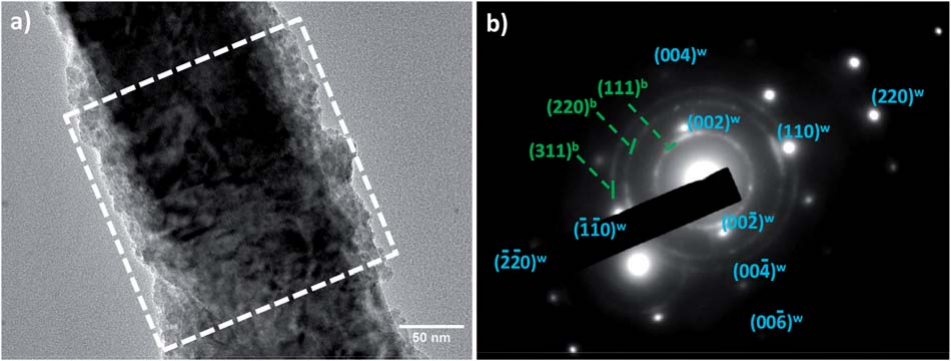
TEM image of one representative ZOS8 rod and its SAED diffraction pattern, consisting of the superposition of the signature of a pseudo-single crystalline wurtzite ZnO phase (in blue) and a polycrystalline blende ZnS one (in green).

Selected Area Electron Diffraction (SAED) performed on a larger ZOS8 sample zone (a square of about 200 × 200 nm^2^) focusing on a single rod minimize this interfacial feature. In other words, they exist but they do not affect the whole ZnO crystalline volume. They remain confined at the ZnO–ZnS interface. Indeed, considering the whole rod volume, a (001) oriented pseudo-single crystalline zinc oxide pattern superposed to a disoriented polycrystalline zinc sulphide one were obtained (Fig. 6).

**Fig. 7:**
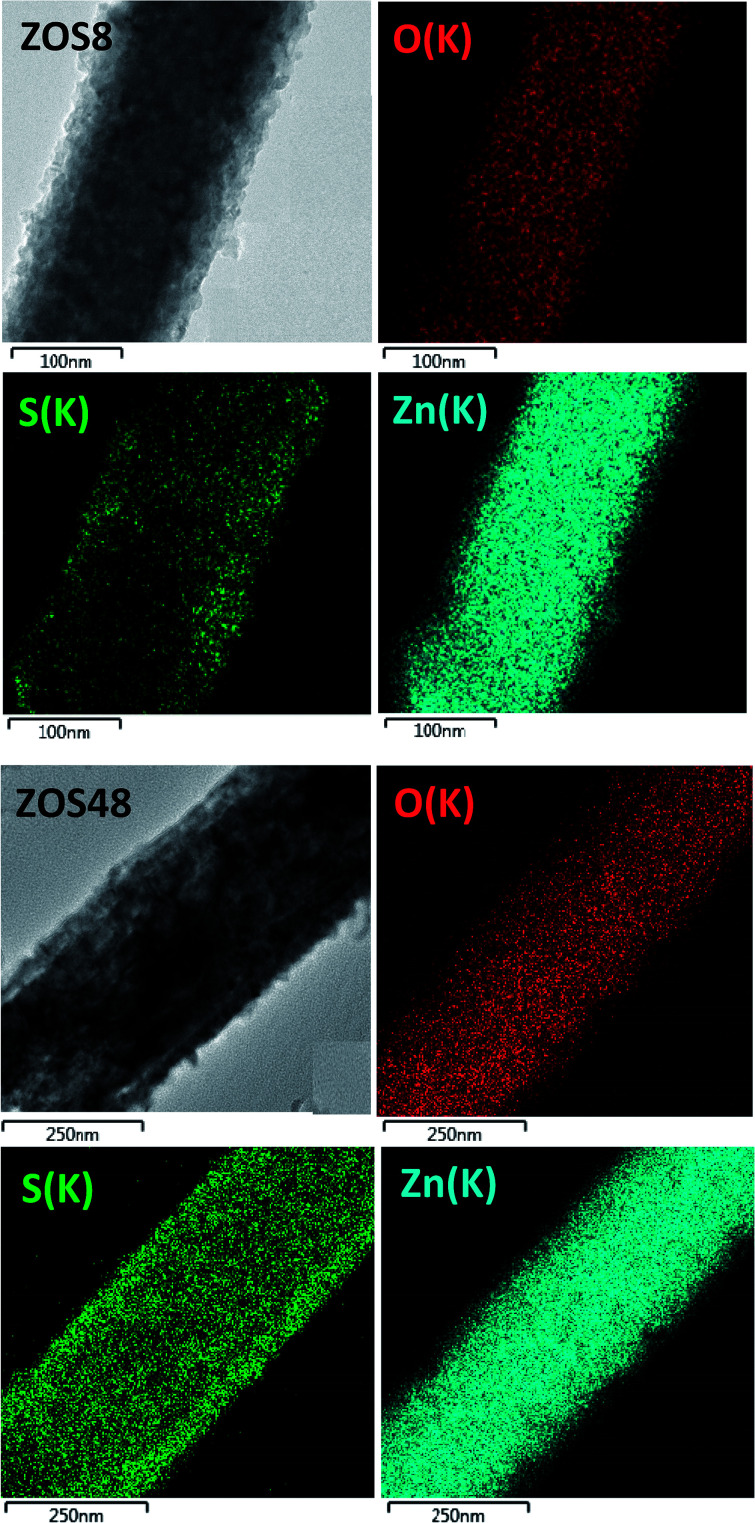
O, S and Zn EDS mapping performed on representative ZOS8 (up) and ZOS48 (down) rods.

**Fig. 8:**
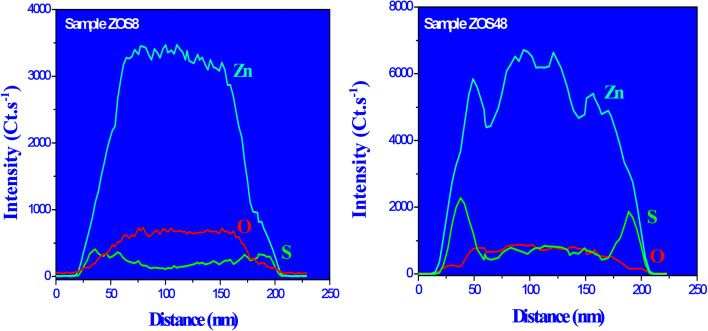
O, S and Zn EDS profile lines recorded across representative ZOS8 (left) and ZOS48 (right) rods.

To complete our structural and microstructural investigations, we performed EDS chemical mapping on some representative composite rods to have more direct information on the distribution of O, S and Zn elements. The obtained images confirmed the core–shell structure (Fig. 7). They also allowed plotting the concentration profile across the rods of O and S elements (Fig. 8) and to estimate from the recovered curves, more accurately the thickness of the ZnS shell. Considering the two extremal peaks of the sulphide profile line, and looking at their basal broadening we measured a zinc sulphide thickness of about 45 ± 3 nm in ZOS58 sample and 23 ± 3 nm in the ZOS8 one, close to that estimated by HRTEM analysis.

**Fig. 9:**
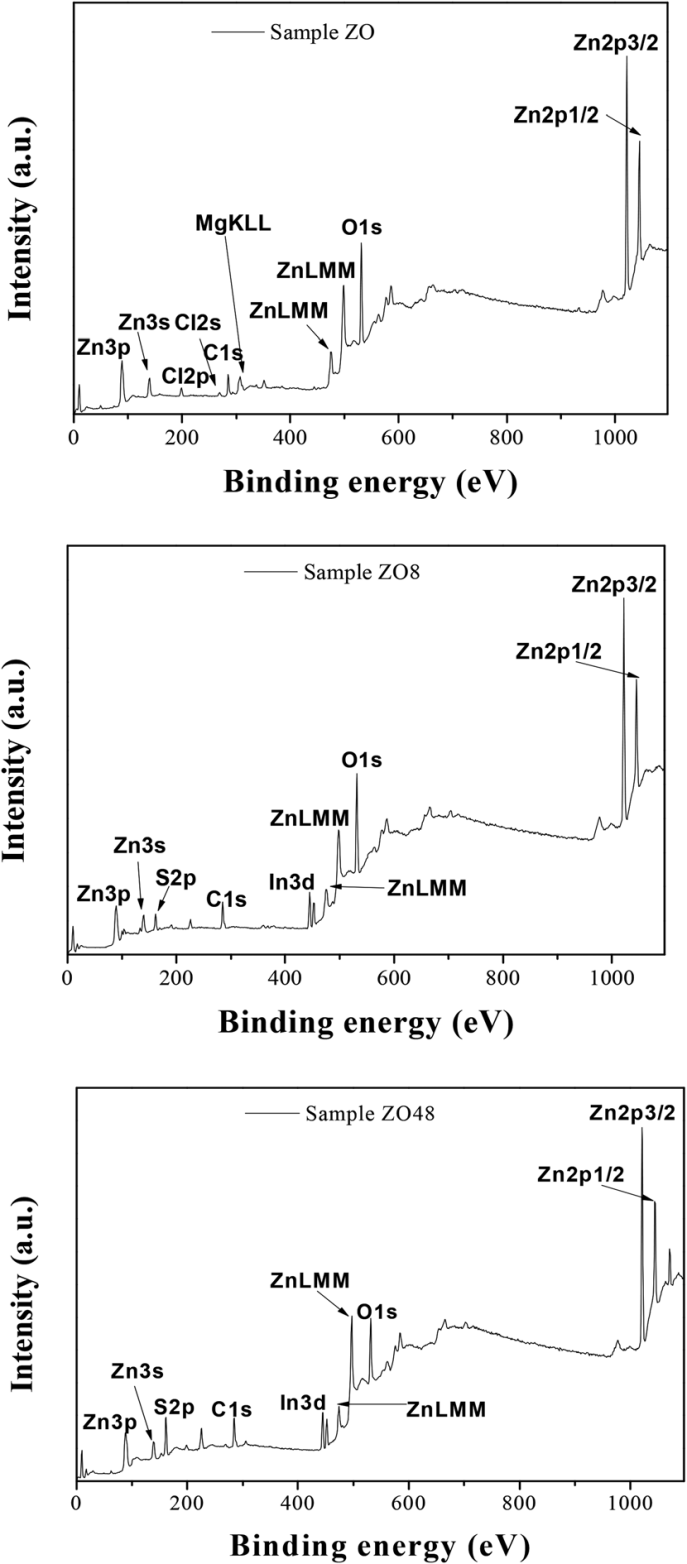
XPS survey spectra of ZO, ZOS8 and ZOS48 samples.

The surface chemical composition of the rods was also checked by X-ray Photoelectron Spectroscopy (XPS). The energy was calibrated against the adventitious carbon main C1s peak at 285 eV. All the survey spectra (Fig. 9) include the signal of Indium (less than 1 at%) confirming that the XPS analysis depth is consistent with the thickness of all the ZnO and ZnO–ZnS on ITO.

They mainly show the signature of O1s and Zn2p at about 530 and 1022 eV, respectively (Table 2), but that of S2p at 162 eV is found only in ZOS8 and ZOS48. Note some contaminants like Cl, K, and Na element can be detected on the recorded spectra: Cl2p at 200 eV, Cl2s at 268 eV, K2p at 293 eV and Na1s at 1072 eV. The former peaks were originated from the ZnO electrodeposition process while the latter was due to the post-sulfidation treatment. These trace elements would not be interfering with the pursued PEC reactions.

**Table 2:** XPS atomic quantification of the nanostructures produced, neglecting the indium contribution in the composites

Film	Signal	*E* (eV)	FWHM (eV)	Area (Cp s^−1^ eV)	at%
ZO	C1s	285.04	1.60	29 314.58	27.40
	In3d	444.61	1.74	7100.11	0.89
	O1s	531.87	2.50	139 513.82	47.01
	S2p	161.67	0.00	0.00	0.00
	Zn2p	1022.17	1.75	371 144.59	24.69
ZOS8	C1s	285.04	1.93	32 610.42	29.71
	O1s	531.87	2.09	115 734.80	38.17
	S2p	161.67	2.31	21 088.13	11.25
	Zn2p	1022.17	2.10	322 406.56	20.86
ZOS48	C1s	285.04	1.55	14 594.49	42.99
	O1s	531.87	2.13	13 823.91	14.59
	S2p	161.67	2.08	14 154.20	18.45
	Zn2p	1022.17	1.64	136 789.95	23.96

**Fig. 10:**
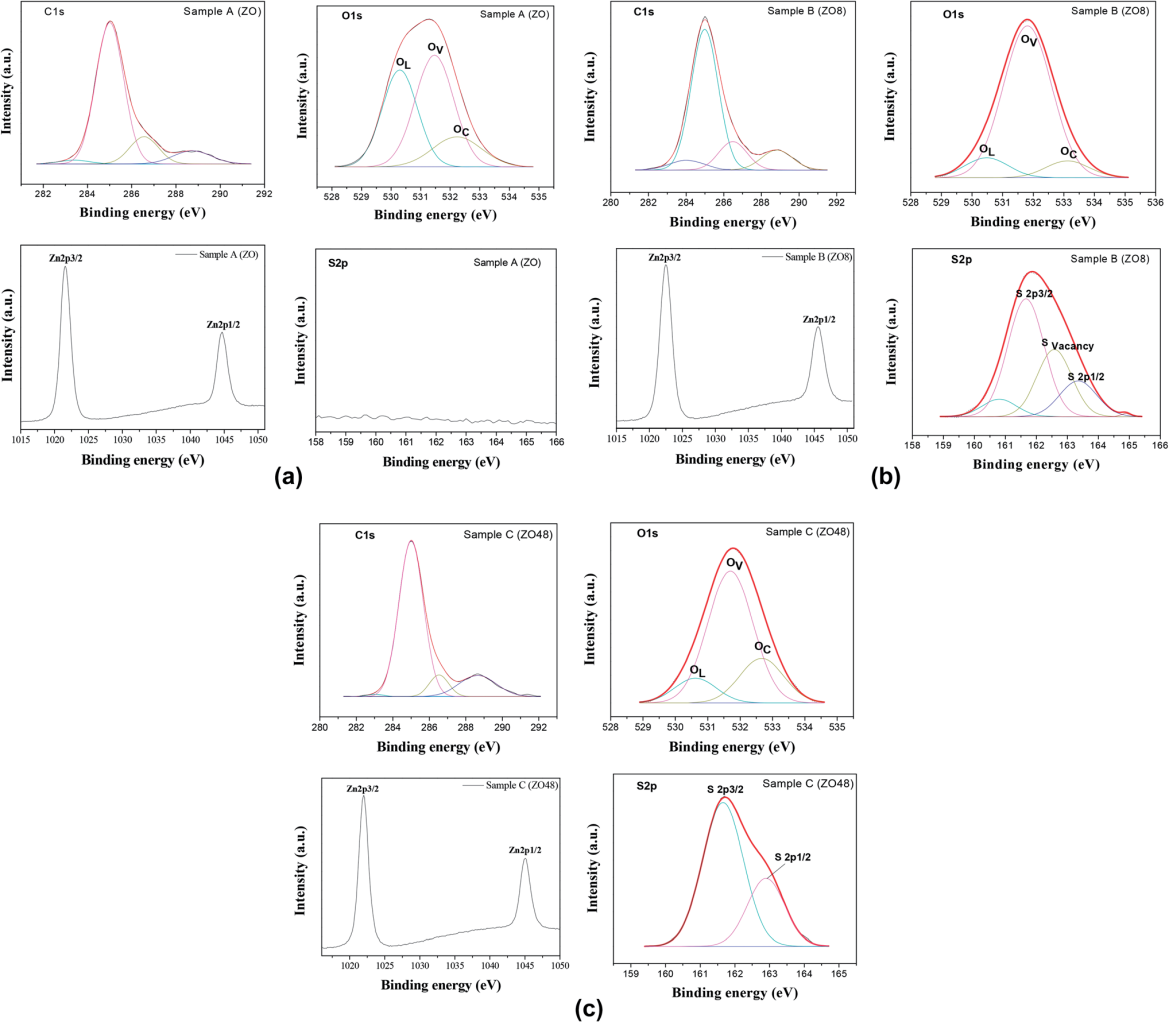
(a) High-resolution XPS spectra of C1s, O1s, Zn2p and S2p recorded on ZO sample. (b) High-resolution XPS spectra of C1s, O1s, Zn2p and S2p recorded on ZOS8 sample. (c) High-resolution XPS spectra of C1s, O1s, Zn2p and S2p recorded on ZOS48 sample.

Moreover, the high resolution spectra of the Zn2p signal of all samples appear to consist of two components attributed to Zn2p_3/2_ and Zn2p_1/2_, their energy positions differing slightly for native ZO and the ZOS8 and ZOS48 composites. They are 1021.69 and 1044.70 eV in ZO (Fig. 10a), similar to the values reported for bulk ZnO,^16^ and shifted to 1022.10 and 1045.10 eV in ZOS8 and ZOS48 (Fig. 10b and c), as in various Zn–S bonds.^17–22^

This finding agrees well with the appearance of the S2p signal in the spectra of these samples (Fig. 10b and c) and its absence in that of ZO (Fig. 10a). This signal is split into two main components of different intensities, a stronger one at 162.01 eV, attributed to S^2−^ anion, and a weaker one at 168.79 eV (see Fig. SI-2 in the ESI†) usually attributed to SO_4_^2−^ anion, as a result of rod surface oxidation. Such a feature is commonly observed in ZnS nanocrystals because of their small size and high surface reactivity.^15,23,24^ The intensity ratio between the S^2−^ and SO_4_^2−^ peaks increases significantly from ZOS8 to ZOS48. It is about 15% in the former and close to 0% in the latter, suggesting that the ZnS layer is less oxidized when its thickness increases. Interestingly, the S^2−^ peak is broadened and slightly asymmetrical in sample ZOS8 mainly. This may result from the overlapping of four contributions: first ones attributed to S^2−^ in a pure ZnS lattice at 161.67 eV (S2p_3/2_) and 163.31 eV (S2p_1/2_) and second ones associated with S^2−^ at the ZnS/ZnO interface at 160.79 eV and 162.62 eV (Fig. 10b). This sulphide anion is labelled as S_O_ in the Kroger–Vink notation and corresponds to a chalcogenide filling a surface ZnO oxygen vacancy.^25^ This feature must be underlined, because it expresses a decrease in the number of oxygen surface defects in ZnO NRs, meaning that the creation of the ZnO–ZnS interface reduces the number of photogenerated charge traps. Interestingly, the sulphide 2p XPS signal of ZOS48 sample was successfully deconvoluted considering only two contributions, those of S2p_3/2_ and S2p_1/2_ peaks at 161.69 and 162.90 eV, respectively (Fig. 10c).

The high-resolution XPS spectra of the O1s signal of all samples were also recorded (Fig. 10) and analysed. Once again, differences between the composites and the parent material appear in the general O1s peak shape. It is broadened, asymmetrical and shifted to lower binding energy (by about 0.10 eV) in ZO compared to ZOS8 and ZOS48. In fact, the O1s signal of native ZnO rods can be decomposed finally into three main components, O_L_ corresponding to O^2−^ ions in the ZnO crystal, O_V_, which is indexed to O^2−^ ions in the oxygen-deprived areas within the ZnO crystal lattice, and O_C_ which represents chemisorbed oxygen species, at 530.29, 531.48 and 532.24 eV, respectively.^26,27^

Moreover, the O/Zn atomic ratio for the ZnO phase in this sample, calculated by deconvoluting the O1s peak and dividing the peak area of the lattice O1s contribution by that of Zn2p_3/2_, is about 0.78, which confirms a net oxygen deficiency. We believe that oxygen vacancies occupy surface positions in the crystal wurtzite lattice, forming native defects.^28^ Clearly, the ZnO surface defect density, mainly oxygen vacancies and oxygen-based adsorbed species, is less in these ZnO–ZnS hetero-nanostructures. The former are filled by S^2−^ anion diffusion and the latter are removed by ZnS shell growth.

### B Optical properties

**Fig. 11:**
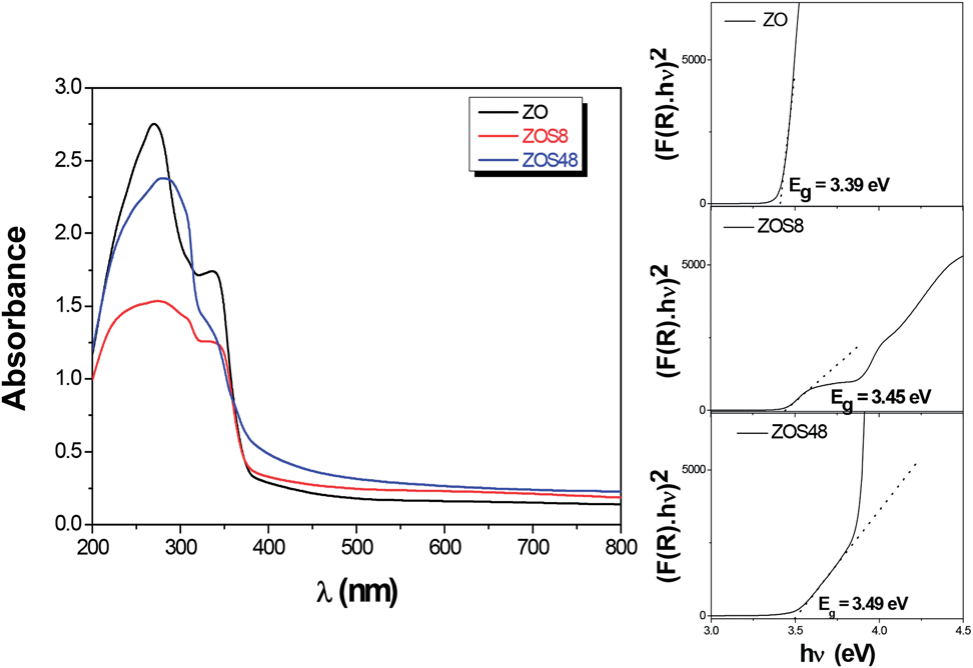
Optical spectra of ZO, ZOS8 and ZOS48 recorded in diffuse reflectance mode. The Tauc plots used to determine the band-gap values are given in inset.

Optical absorption spectroscopy (OAS) was performed and the collected spectra on ZOS8 and ZOS48 samples, in the diffuse reflectance mode, were compared to that obtained on pristine ZO one (Fig. 11).

It is well-known that the optical absorption properties of any processed material are strongly dependent on its structure and microstructure. The sulfidation treatment, beside adding a second semiconductor to the initial ZnO phase, creates junctions, changes the surface states, affects the crystallographic quality of the native material, and consequently, changes its optical response more or less significantly. In the present case, in the UV region, between 300 and 400 nm, all the spectra show a strong absorption typical of ZnO and ZnS wide band-gap semiconductors (Fig. 11). The gap values inferred from these data using the Tauc plots (see the inset in Fig. 11) are typically between the reference values of bulk ZnO and ZnS. They are of 3.39, 3.45 and 3.49 eV for ZO, ZOS8 and ZOS48, respectively, the gap increasing with the ZnS shell thickness. In the visible region, between 400 and 800 nm, there is a wide but weak absorption band. The origin of this band differs from one sample to another. It is due to surface ZnO defects, which act as radiative recombination sites in ZO, while it is attributed to a type-II interfacial transition between the ZnS shell and the ZnO core in ZO8, as already established by room temperature photoluminescence (PL) measurements (see Fig. SI-3 in the ESI†).^11^ There are two main differences between the PL spectra of ZO and ZOS8, firstly, a net decrease in the whole PL intensity for ZOS8 proves that there is less radiative recombination. Moreover, the orange emission observed for ZO, attributed to oxygen vacancies in native ZnO NRs, disappears in ZOS8 as a consequence of their filling by S atoms.^11^

**Fig. 12:**
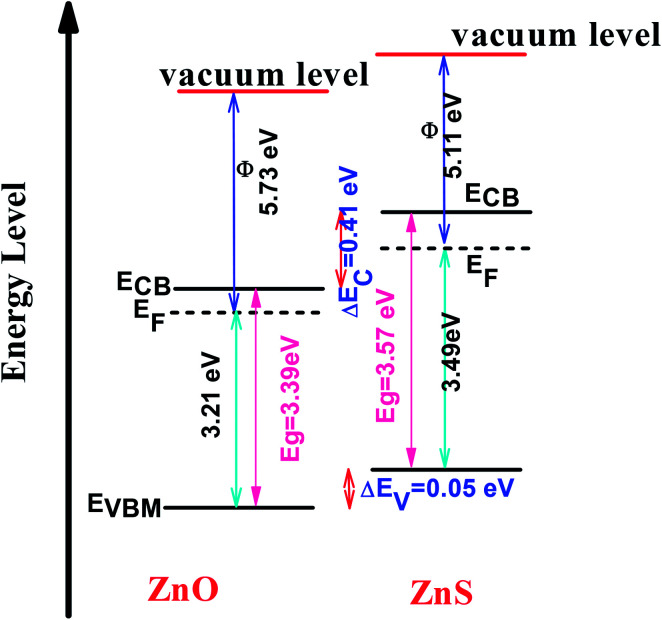
Band alignment diagram of ZnO–ZnS heterojunction.

These optical investigations were completed by performing UV-visible photoelectron spectroscopy (UPS) and OAS measurements on two ZnO and ZnS, supported on ITO, reference samples (see Fig. SI-4 in the ESI†). The ZnO reference was prepared in the same conditions than ZO sample, while the ZnS one was obtained, starting from a very thin ZnO film deposited on ITO by electrodeposition (ZnCl_2_ (5 mM) and KCl (1 M)) applying a current of −1.3 mA for 300 s. A sulfidation treatment was then performed in Na_2_S solution (0.32 M) for 48 h, to transform almost completely the initial zinc oxide film into a zinc sulphide one. These data were used to determine the relative valence and conduction band positions of each semiconductor and to draw the band alignment diagram of ZnO/ZnS heterojunction (Fig. 12) to confirm the expected charge transfer from one semiconductor to another.

### C Photo-electrochemical properties

**Fig. 13:**
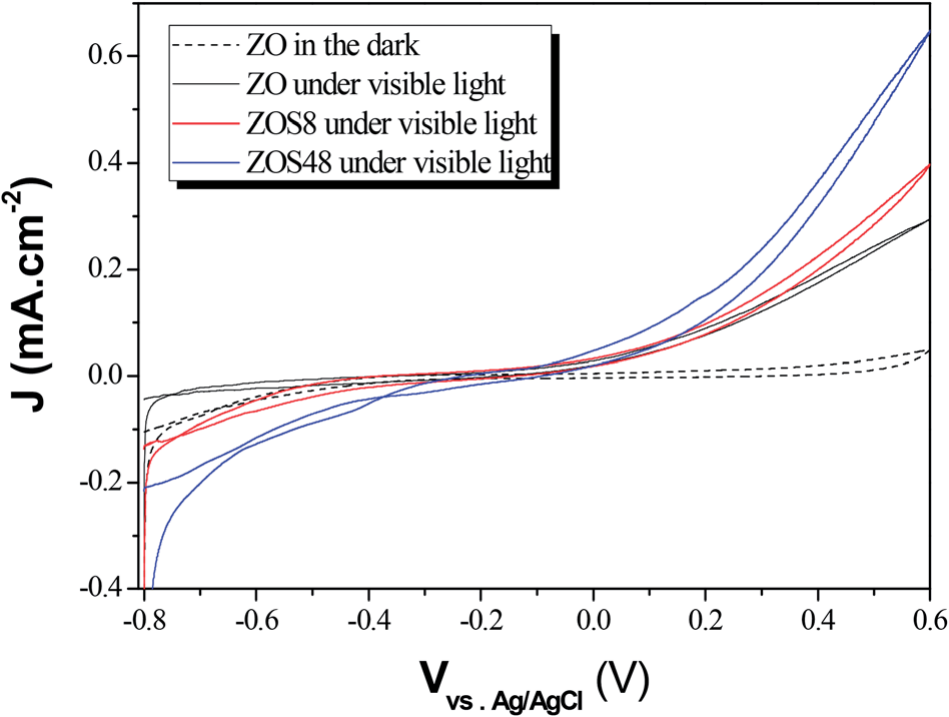
Photocurrent *vs.* potential curves plotted under dark and simulated sunlight for ZO, ZOS8 and ZOS48 samples (scan rate = 10 mV s^−1^).

The PEC performances of ZO, ZOS8 and ZOS48 were investigated by running cyclic voltammetry in the dark and under simulated solar light in the −0.8 to 0.6 V potential range (Fig. 13). As expected, all samples show negligible current in the dark. When illuminated, all exhibit a non-zero current densities, but the value is significantly higher for ZOS8 and ZOS48 than ZO, due to synergetic effects in charge generation, separation and transport in the former structures. It should be noted that flatband potential of these hybrids is lower than that of the parent material, meaning that the energy barrier is lower in heterojunctioned ZnO NRs than in pure ones.^29,30^ When the applied bias potential is increased, *J*_p_ increases, the highest values being reached at *V* = 0.6 V.

Using these data, namely *J*(*V*) under illumination, we calculated the solar conversion efficiency *η* for each photoanode, separately from the other half of the water splitting reaction, and its related fill factor ff, using the following equations:^31^1
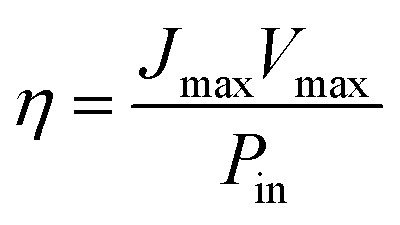
2
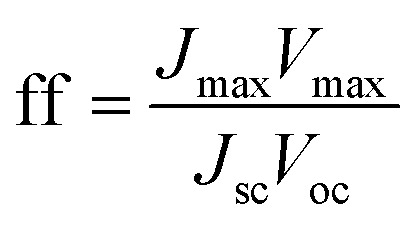
where *V*_max_ and *J*_max_ are respectively the voltage and the current density at the maximum power point, *P*_in_ is the power density of the illumination (in W cm^−2^) and *V*_oc_ and *J*_sc_ are respectively the open circuit voltage and the short-circuit current density. The obtained values were summarized in Table 3.

**Table 3:** Main photoelectrochemical characteristics of the engineered photoanodes, using a xenon lamp and working in a three electrodes PEC configuration

	*J*_sc_ (mA cm^−2^)	*V*_oc_ (V)	*J*_max_ (mA cm^−2^)	*V*_max_ (V)	ff	*η*
ZO	−0.371	0.028	−0.142	0.011	0.145	0.031
ZOS8	−0.371	0.028	−0.142	0.013	0.179	0.038
ZOS48	−0.047	0.229	−0.095	0.019	0.165	0.036

As expected, the measured photoanode efficiency and ff factor were found to be higher in the ZnS coated rods than in the native ZnO ones. Interestingly the differences between those measured on the thinly and thickly coated rods were weak, suggesting that the photocurrent increase observed in the latter composite sample did not traduce a better efficiency toward PEC application. In one hand, the photogenerated by the ZnS semiconductor have to cross a longer matter section to reach the ZnO semiconductor core before being injected into the external circuit and in the other hand, its inherent microstructural defects (interfacial discontinuities, gaps and porosities) act as charge transfer barriers, limiting their transfer to the ZnO band conduction.

**Fig. 14:**
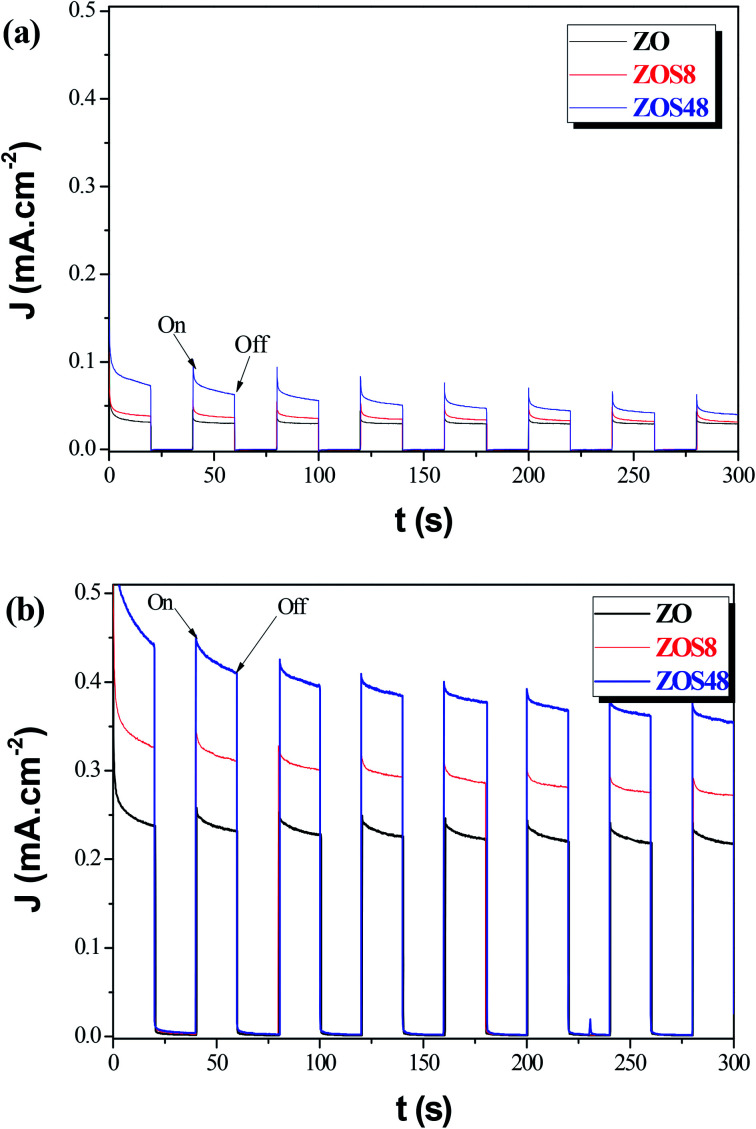
Photocurrent responses in Na_2_SO_4_ (0.5 M, pH 7) of ZO, ZOS8 and ZOS48 samples under xenon lamp illumination at (a) 0 and (b) 0.5 V.

Whereas, these results agree with previous studies which demonstrate that the surface functionalization of ZnO with ZnS creates novel materials for photovoltaic and photocatalytic applications,^14^ they also underline the importance of their final microstructure. It affects their efficiency but also, it may affect their chemical stability. So, we decided to test the reproducibility of the photoresponse of our photoanodes, by measuring their transient photocurrent in 0.5 M Na_2_SO_4_, under intermittent illumination with and without bias potential (namely, 0 and 0.5 V *vs.* Ag/AgCl). The obtained curves were plotted in Fig. 14a and b, respectively. An instantaneous change in current upon illumination for all the photoanodes was observed. The current returned to the original values almost immediately the light was switched off. Initially, the ZnS shell seems to improve the ZnO PEC response. Typically it significantly increases the initial photocurrent density, (*J*_p_)_ini_ from 0.05 (ZO) to 0.10 mA cm^−2^ (ZOS8) and 0.15 mA cm^−2^ (ZOS48) at 0 V, and from 0.39 to 0.45 and 0.60 mA cm^−2^ at 0.5 V, respectively. Under chopped Xe lamp irradiation, the heterostructures exhibit a prompt photocurrent response. The increase in the photocurrent when a bias is applied is a key feature for the realization of a type-II heterostructure separating the electrons and holes in the composites. Unfortunately these improvements are not stable over time and current fluctuations appear after only 5 minutes of operation. In this time the photocurrent density of ZO, ZOS8 and ZOS48 decreases from its initial value by about 8, 15 and 45%, respectively, in a passive electrolyte. In the absence of scavengers (we voluntarily do not put into the electrolyte solution sacrificial S^2−^ and/or S_2_O_3_^2−^ ions, whose oxidation potentials are significantly higher than that of the anodic ZnS decomposition), the photoanodes start to decompose rapidly, and this decomposition seems to be more important in ZOS48 than in ZOS8 and ZO because of its surface microstructure. Finally, the best compromise is offered by the ZOS8 sample. Its thin and continuous ZnS coating makes its PEC properties comparable to those previously reported for differently prepared ZnO–ZnS heterostructures (Table 4). This sample clearly exhibits (i) improved absorption in visible light, (ii) reduced charge trapping probability, (iii) fast and efficient transfer of photogenerated charges, and (iv) a photo-degradation ratio comparable to that of pure ZnO.

**Table 4:** Photocurrent density measured on differently prepared ZnO–ZnS heterostructures, working at 300 K in a three-electrode PEC cell, using a Pt cathode and a Ag/AgCl reference electrode

PEC conditions	Material processing	Morphology	*J*_initial_ (mA cm^−2^)
0.5 M Na_2_SO_4_/0.1 M Na_2_S, xenon lamp (100 W), light exposed area not indicated	ZnO produced by hydrothermal route followed by sulfidation in 1 M Na_2_S solution^12^	ZnO nanowires of ∼250 nm in diameter coated by a thin ZnS layer (20–30 nm)	0.35 (at 0.5 V)
0.1 M Na_2_SO_4_, xeon lamp (100 W), light exposed area of 0.785 cm^2^	ZnO produced by hydrothermal route followed by hydrothermal treatment in 0.016 M Na_2_S_2_O_3_ solution^32^	ZnO multipods of 1–2 μm in thickness coated by a thin ZnS layer	0.10 (at 0 V)
0.1 M Na_2_SO_4_, xenon lamp (200 W), light exposed area not indicated	ZnO produced by hydrothermal route followed by sulfidation in Na_2_S solution^23^	ZnO nanorod arrays of 100–200 nm in diameter coated by a thin ZnS layer (20 nm)	0.38 (at 0.9 V)
0.5 M Na_2_S_2_O_3_, solar simulator (100 mW cm^−2^), light exposed area of 0.785 cm^2^	ZnO produced by hydrothermal route followed by sulfidation in 0.05 M Na_2_S solution^30^	ZnO nanorod arrays of ∼200 nm in diameter coated by a thin ZnS layer of different thicknesses	0.98–1.61 (at 0.1 V)
0.1 M Na_2_SO_4_, xenon lamp (150 W), light exposed area of 0.70 cm^2^	ZnO produced by electrodeposition followed by sulfidation in 0.32 M Na_2_S solution^a^	ZnO nanowires of ∼100 nm in diameter coated by a thin ZnS layer of 20–50 nm in thickness	0.45–0.60 (at 0.5 V)

a This work.

These results underline the importance of the material processing conditions on the microstructural properties of the engineered electrodes and on their ability to be used as efficient PEC photoanodes. They also underline the fact that the processing conditions act directly on the ZnS shell growth mechanism and how this mechanism can make the resulting electrode more or less stable toward photochemical corrosion.

### D Crystal growth mechanism

**Fig. 15:**
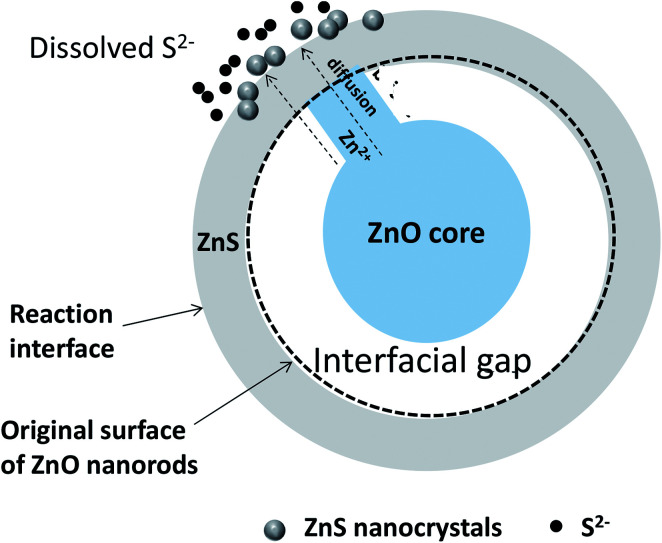
Schematic sulfidation mechanism of ZnO NRs immersed in a concentrated aqueous Na_2_S solution.

The structural, microstructural, optical and photo-electrochemical analyses of the produced ZnO–ZnS/ITO samples allowed us to propose a general mechanism for the growth of ZnS crystals around the native ZnO NRs during sulfidation treatment. This mechanism is time-dependent and it changes when the contact time is long. We believe that the conversion from ZnO NRs to ZnO–ZnS core–shell NRs involves diffusion and consumption reactions. In the initial stage, Na_2_S dissociates in water. The S^2−^ ions formed start by filling-up the surface oxygen vacancies of the ZnO wurtzite lattice. Then, they react with Zn^2+^ slowly dissolved from the surface of the ZnO NRs and form an initial ZnS crystalline shell. The driving force of Zn^2+^ diffusion is caused by the difference of solubility in water of ZnO and Zns solids. ZnS has a lower solubility than ZnO. Small gaps appear between the ZnO cores and initial ZnS shells as ZnO on the surface is gradually converted into ZnS. Meanwhile, part of the ZnO in contact with the ZnS shells serves as convenient transportation pathway (Fig. 15) for Zn^2+^ ions to reach the reaction interface *via* surface and bulk diffusion processes. Then zinc ions continuously diffuse from inside the ZnO core to the outer surface of the ZnS shell along these diffusion pathways. It is easier for Zn^2+^ than S^2−^ to pass through the ZnS shells, due to the smaller size of Zn^2+^, and to react with S^2−^ in solution. Thus, ZnS shells become thicker as more and more ZnS nanocrystals pile up on the initial ZnS shells. Note that some ZnS nanocrystals do not act as building units of ZnS shells but remain in the reaction liquid medium. As conversion proceeds, the interfacial void is enlarged due to the continuous consumption of ZnO cores, and ZnO–ZnS core–shell nanocomposites are obtained correspondingly. The material characterisation results confirm that solid–solid diffusion of anions and cations proceeds in the chemically modified surface zone and around it.

Noteworthy, the joint dissolution co-precipitation process consists of several sequential steps, which can be summarized in the following way:^34^ (i) arrival of the dissolved species to the vicinity of the inorganic surface (S^2−^ in our case) and, in parallel, (ii) the dissolution of the inorganic substrate (Zn^2+^ in our case), which causes (iii) a change in the chemistry of the inorganic crystal-aqueous solution interface, mainly an increase in the concentration of certain aqueous species, which in turn changes the solution pH. This can lead to the (iv) increase in supersaturation of the aqueous solution with respect to a secondary phase that may result in (iv) the precipitation (heterogeneous nucleation) of a more or less adherent precipitate which consists of chemical species from both the aqueous solution (S^2−^ in our case) and the dissolution of the inorganic substrate (Zn^2+^ in our case).

As one might expect, the cited above interfacial phenomena depend crucially on the native substrate-surface topography but also on its evolution over the time. Indeed, the presence of water alters the entire surface structure through dissolution processes, leading to the formation of a kind of surface altered layer. So the precipitation occurs while the surface is dissolving, and it is enhanced by the appearance of dissolution-induced surface microdefects (vacancies, etch pits, microfractures).^35^ Paradoxically, this porosity is critical for the access of fluids to the reactive surfaces during replacement reactions and the advancement of the reaction boundary. It also may be transient feature and can be at least partially resorbed, working at higher temperature (very close to the water boiling point), and/or for a very long time (several days) to reach a kind of textural equilibration, a form of Ostwald ripening. It must be underlined that these erasing processes are not total and a residual porosity may remain even at the nanometer scale.^36^

The growth mechanism of the product phase is also a key variable to be considered during the analysis of porosity generation in replacement reactions. Intuitively, one may accept that if a good epitaxial fit, formed by some crystallographic continuity, exists between the parent and product phases, the product will grow as a thin film homogeneously covering the parent phase. In contrast, if the structure of the product is appreciably different from that of the substrate, like in the case of ZnO and ZnS solids, it will tend to precipitate by three-dimensional heterogeneous nucleation.^37^ The distribution of the precipitated volume will be different in these two opposite cases, and while in the first case, full coverage of the substrate surface may be achieved, in the second case pore space will be maintained at the interface between the parent phase and the product, which is what we progressively observed by building our ZnO–ZnS heterojunctions.

Additional, the solid phase (ZnS in our case) overgrowing on the dissolved substrate surface (ZnO) forms aggregates supported on the substrate surface. Longer periods of interaction lead to the formation of aggregates that consist of scattered tufts with well-developed but non-oriented crystals. As a consequence a rough and less-defined surface shape would characterize the final morphology of the treated material. Obviously, the lack of structural relationships between substrate and the deposit favors the formation of a discontinuous surface layer composed of more or less well-shaped deposit nanocrystals oriented randomly with respect to the substrate, marking even more the rough nature of the final surface.^38^

## Experimental

### A Sample preparation

All chemicals were of analytical reagent grade and were used as received without further purification. All aqueous solutions were prepared using deionized water.

**Fig. 16:**
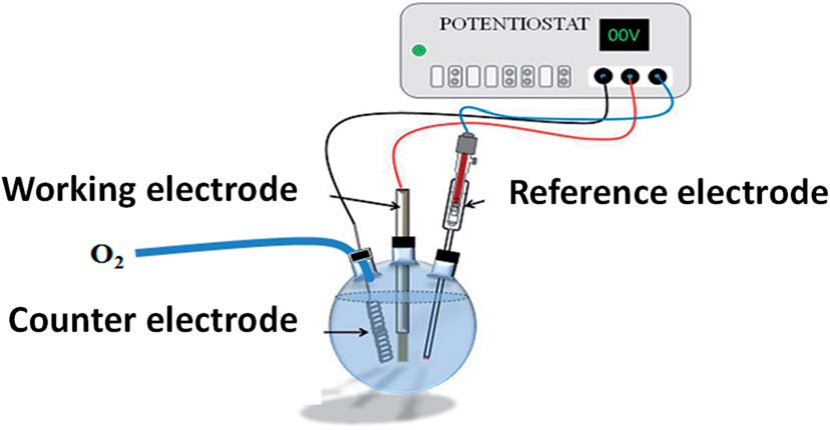
General scheme of the electrochemical cell used for ZnO NR electrodeposition on ITO sheet (working electrode).

**Fig. 17:**
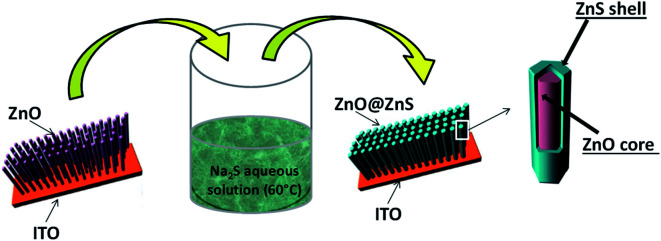
General scheme for ZnO NR coating by a continuous nanocrystalline ZnS shell.

As previously reported,^11,15^ ZnO NRs, fairly well aligned, and perpendicular to the ITO surface, were produced by electrodeposition (Fig. 16). Cleaned ITO sheets (resistance 10 Ω m) were immersed in a ZnCl_2_ (0.5 mM) and KCl (0.1 mM) aqueous solution, under continuous bubbling of dioxygen, and used as working electrodes in a classical 3-electrodes electrochemical cell under optimized potentiostatic conditions.^11^ A potential of −1.0 V *vs.* Ag/AgCl was applied for 9000 s at 80 °C. The as-prepared ZnO/ITO sample was then immersed in an aqueous solution of 3.2 × 10^−1^ M of Na_2_S, and placed in a 60 °C water bath for 8 h and 48 h (Fig. 17). The samples were then washed with water and absolute ethanol before drying in air at 80 °C for 12 h.

### B Sample characterization

XRD analysis was carried out by means of an Empyrean (PANALYTICAL) diffractometer equipped with a multichannel PIXcel 3D detector and a Cu Kα X-ray source (1.5418 Å). Typically, each pattern was recorded under grazing conditions (GXRD) in the 15–80° 2*θ* range (0.06° for 1.760 s). A 1/16 divergence slit was installed in front of the sample to form a quasi-parallel incident beam. Note that the samples are mounted on a five-axis cradle with motorized movements to obtain perfect horizontality. This cradle also allows to apply a phi-rotation (around the *z*-axis) and a psi-tilt (around the *x*-axis). X-ray-based texture analysis was performed using the same equipment, by employing poly-capillary optics for the incident beam and installing a collimator directly in front of the detector.

SEM and TEM were performed using Supra40 ZEISS and JEOL JEM-100CX-II microscopes operating at 5 and 100 kV, respectively. HRTEM was performed on suspensions containing the nanorods, sonicated for few minutes in ethanol before deposition of a few drops on copper-coated carbon grids. HRTEM experiments were performed on a JEOL JEM 2010 UHR microscope operating at 200 kV. The images were collected with a 4008 × 2672 pixel CCD camera (Gatan Orius SC1000). Chemical analyses were obtained by an EDS microanalyser (PGT-IMIX PC) mounted on the microscope. Additionally, high-angle annular dark-field scanning TEM (HAADF-STEM) and EDS X-ray microanalysis for chemical mapping were carried out on a JEOL JEM 2100Plus TEM microscope, operating at 200 kV, interfaced to Oxford Instruments AZtec EDS system with an X-Max T large area (80 mm^2^) SDD detector.

XPS was performed using a Thermo VG ESCALAB 250 instrument equipped with a micro-focused, monochromatic A1 Kα X-ray source (1486.6 eV) and a magnetic lens. The X-ray spot size was 500 μm (15 kV, 150 W). The spectra were acquired in the constant analyser energy mode with pass energies of 150 and 40 eV for the general survey and the narrow scans, respectively.

Finally, the UV-visible diffuse reflectance spectra of the samples were recorded in the 200–800 nm range on a Perkin Elmer-Lambda 1050 spectrophotometer equipped with a PTFE-coated integration sphere.

### C Photoelectrochemical measurements

Photoresponses were evaluated by measuring the photocurrent density *J*_p_, using a VMP100 potentiostat from Bio-Logic. *J*_p_ was measured as a function of the applied potential *E* in a standard three-electrode (single-compartment) home-made cell. The potential of each sample (used as working electrode) was measured using a Ag/AgCl electrode and a Pt wire counter-electrode in a solution of Na_2_SO_4_ (0.5 M, pH 7). The whole cell was purged with argon prior to all experiments. A sample area of 0.7 × 1.0 cm^2^ was illuminated by a 150 W xenon lamp (ORIEL instruments), to mimic solar light, leading thus to an incident light power density *I*_0_ of 50 mW cm^−2^.

## Conclusions

ZnS coating of vertically aligned ZnO NR arrays can be easily achieved by chemical sulfidation using aqueous Na_2_S. The ZnS shell formed has a polycrystalline cubic blende structure with a thickness which increases with the sulfidation treatment time. This important material processing parameter affects the roughness of the resulting heterostructures and the crystallographic quality of the ZnO–ZnS interface. Partial disorientation of native ZnO crystallites during ZnS formation is observed with the formation of interfacial voids when the sulfidation is prolonged. As a consequence, in PEC experiments the observed improvement of the initial current density in the heterostructures compared to the native material, decreases rapidly with time. Typically, using a passive electrolyte, the current fluctuation reaches 45% over only 5 minutes of operation, under Xe lamp illumination, in the photoanode produced with a long treatment time, while it does not exceed 15% in that prepared with a short one. These results demonstrate the importance of this material processing parameter in elaborating more or less efficient photoanodes for PEC applications.

## Conflicts of interest

There are no conflicts to declare.

## Supplementary Material

RA-008-C8RA00176F-s001
