# Supplementary material for: The structural and the photoelectrochemical properties of ZnO–ZnS/ITO 1D hetero-junctions prepared by tandem electrodeposition and surface sulfidation: on the material processing limits
Source: RSC Adv. 2018 Mar 27;8(21):11785–98. doi: 10.1039/c8ra00176f (PMC9092361; doi:10.1039/c8ra00176f)
Supplement: RA-008-C8RA00176F-s001 [file RA-008-C8RA00176F-s001.pdf]

Electronic Supplementary Material (ESI) for RSC Advances.
This journal is © The Royal Society of Chemistry 2018

The structural and the photoelectrochemical properties of ZnO-ZnS/ITO 1D hetero-junctions prepared by tandem electrodeposition and surface sulfidation: on the material processing limits

Supporting information

Textural analysis

X-ray pole figures measured on a Panalytical Empyrean diffractometer system, within a quasi-parallel incident beam configuration, for the (101), (102) and (103) ZnO crystallographic plans, exhibit maximal intensities for χ-tilt values corresponding more or less to the angles between these plans and the (002) ones (Fig. SI-1).

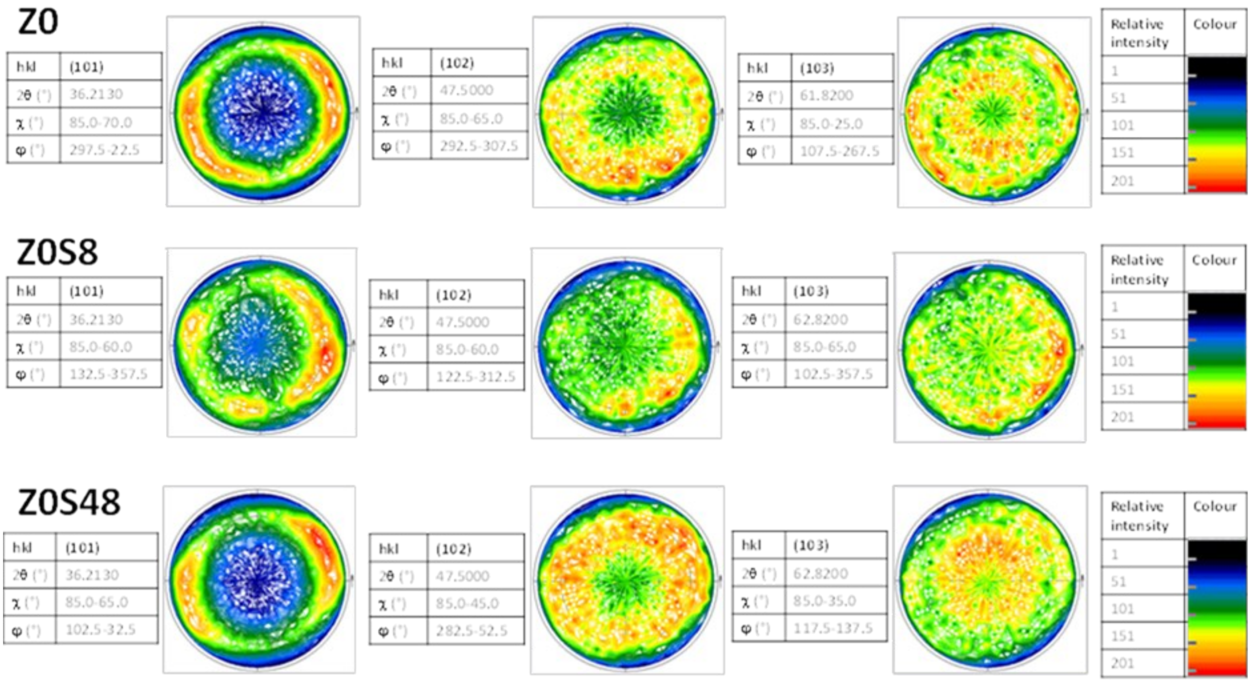

Figure SI-1. X-ray pole figure of ZnO-ZnS nanostructures, corresponding to ZOS8 and ZOS48 samples, compared to those of their ZnO parents (ZO sample) for the (101), (102) and (103) crystallographic plans of the wurtzite lattice.

XPS analysis

More details are given in Fig. SI-2 to illustrate the chemical nature of S element at the surface of the produced ZO, ZOS8 and ZOS48 samples.

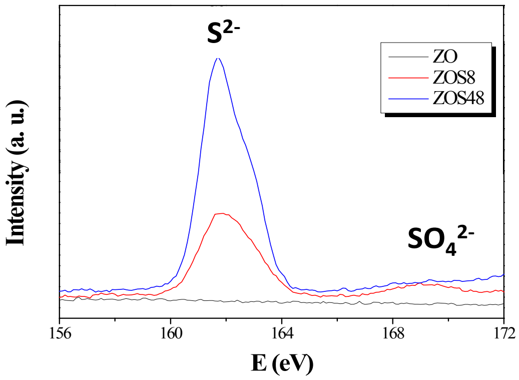

Fig. SI-2. High resolution S2p XPS spectra of ZO, ZOS8 and ZOS48 samples, recorded in the 156-172 eV binding energy range.

Opto-electronic analysis

PL spectra of the produced samples were recorded at room temperature using a PL fluorescence spectrometer (Perkin Elmer LS55) equipped with a Xe lamp (excitation wavelength of 315 nm). They were plotted using Origin8.0 software and compared to evidence any change on the optical response of the ZnO nanorods before (ZO) and after their sulphidation, mainly their sulphidation for a relatively short time (ZOS8). Clearly the PL intensity decreases after rod's treatment (Fig SI-3), and its decrease is more pronounced for the peaks attributed to electronic processes involving oxygen vacancy states.

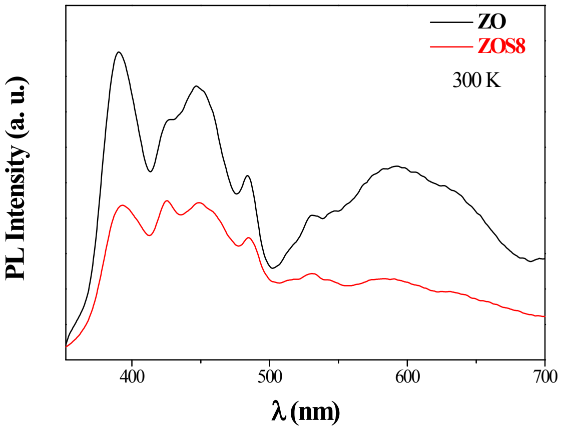

Figure SI-3. The photoluminescence spectra of ZO and ZOS8 samples recorded at 300 K. [11]

Additionally, UPS spectra of two ZnO and ZnS, supported on ITO, references, were recorded, using a AXIS Ultra DLD (KRATOS Inc.) spectrophotometer, equipped with a He I (21.2 eV) UV source and operating under vacuum (4.0 10-8 Torr) with a pass energy of 5 eV (Dwell time = 100 ms, step = 0.02 eV/step) for a typical sample analysis area of 110 µm. The measurement was carried out by applying a bias of -15 V. The data inferred from these spectra and from the optical absorption spectroscopy were used to determine the relative valence and conduction band positions of each semiconductor (Fig. SI-4).

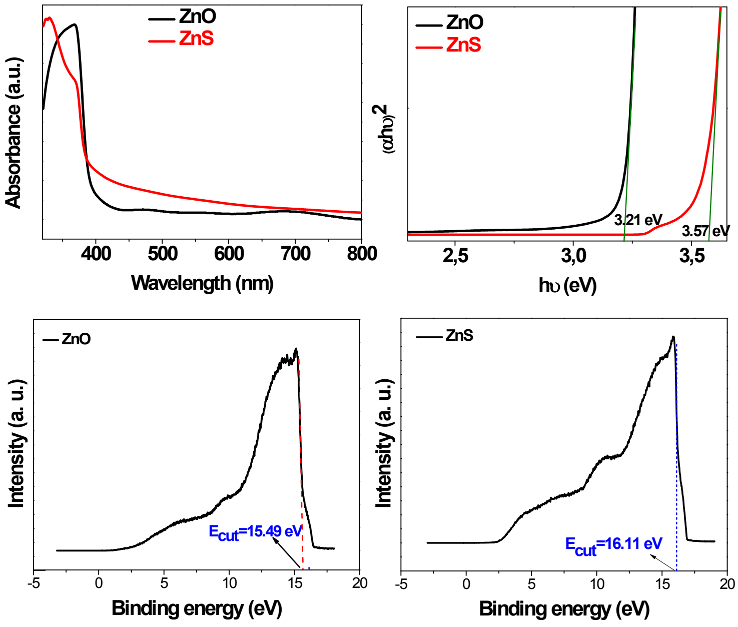

Figure SI-4. Optical absorption spectra recorded within the diffuse reflectance mode of ZnO and ZnS reference samples (up) and their Tauc plots. Their UPS spectra recorded for an applied bias of -15 V (down).
